# Expression and phosphorylation of stathmin correlate with cell migration in esophageal squamous cell carcinoma

**DOI:** 10.3892/or.2012.2157

**Published:** 2012-11-28

**Authors:** FEI LIU, YU-LIN SUN, YANG XU, FANG LIU, LI-SHUN WANG, XIAO-HANG ZHAO

**Affiliations:** 1 State Key Laboratory of Molecular Oncology, Cancer Institute and Hospital, Chinese Academy of Medical Sciences and Peking Union Medical College, Beijing 100021; 2 Beijing Genomics Institute, Chinese Academy of Sciences, Beijing 101300; 3 Center of Basic Medical Sciences, Navy General Hospital, Beijing 100048, P.R. China

**Keywords:** stathmin, esophageal squamous cell carcinoma, migration, phosphorylation

## Abstract

Microtubules play extensive roles in cellular processes, including cell motility. Stathmin is an important protein which destabilizes microtubules. The essential function of stathmin is closely associated with its phosphorylation status. Stathmin is overexpressed in many human cancers and has a significant relationship with clinical characteristics such as grade, tumor size and prognosis. We demonstrated that stathmin was overexpressed in ESCC tissues using both 2-DE and immunohistochemistry analysis. In addition, overexpression of stathmin was significantly correlated with histological grade in ESCC. However, no correlation was found with age, gender and lymph node metastasis. Knockdown of *stathmin* with siRNA impaired cell migration in KYSE30 and KYSE410 cells. When EC0156 cells were treated with paclitaxel, stathmin was stably phosphorylated and migration was impaired. These observations suggest that stathmin may have a more important function in ESCC development and migration. The present study provides further understanding of the importance of stathmin in ESCC therapy or diagnosis.

## Introduction

Esophageal cancer (EC) is the eighth most common cancer worldwide (1), while esophageal squamous cell carcinoma (ESCC) is the predominant histological subtype in Asia, especially in China. It is characterized by high incidence and mortality rate (1,2). We found that stathmin is a differentially expressed protein between cancer and adjacent normal tissues in ESCC using proteomic technology.

Stathmin is an important protein which destabilizes microtubules (3,4). Microtubules are essential for many cellular processes, including mitosis, intracellular transport, supportment of cell shape and cell motility, and stathmin playes an important role in the regulation of microtubule which was involved in the construction and function of the mitotic spindle (5). Phosphorylation of stathmin led to a loss of the microtubule-destabilizing activity (6–8). Inhibition of stathmin phosphorylation produced strong mitotic phenotypes characterized by disassembly and disorganization of mitotic spindles and abnormal chromosome distributions (6). Stathmin phosphorylation gradient was necessary for correct spindle formation. Gradients of diffusible morphogens are known to be crucial for the supracellular self-organization of tissues and organisms (9).

Many studies had reported that stathmin was overexpressed across a broad range of human cancers, including acute leukemia, lymphoma, neuroblastoma and ovarian, prostate, breast and lung cancer (10–12).

The upregulation of stathmin in ESCC was reported and associated with differentiation degree, lymph node metastasis, invasive depth and TNM stage (13,14). Wang *et al* demonstrated that knockdown of stathmin by antisense oligonucleotide can inhibit the proliferation of ECa109 cells (15). The expression and exact biological function of stathmin in ESCC, especially motility, remained largely unclear.

## Materials and methods

### Cell culture

ESCC cell lines EC0156 was established by our laboratory (16). KYSE30, KYSE140, KYSE150, KYSE170, KYSE180, KYSE410 and KYSE510 were donated by Dr Y. Shimada. EC0156 was cultured in Dulbecco’s modified Eagle’s medium (HyClone, UT, USA) supplemented with 10% fetal calf serum, 100 U/ml penicillin and 100 μg/ml streptomycin (Gibco, NY, USA). The other cell lines were cultured in RPMI-1640 medium supplemented with 10% heat-inactivated fetal bovine serum. All the cells were incubated at 37°C in a humidified atmosphere of 5% CO_2_.

### Tissue specimens

From January 1999 to 2002, 8 pairs of specimens for two dimensional electrophoresis were obtained from surgically resected ESCC tissues in Cancer Hospital of Chinese Academy of Medical Sciences (CAMS). Another 50 tissues specimes for immnohistochemistry were also obtained from surgically resected esophageal carcinoma in Cancer Hospital of CAMS from January 1999 to 2009. Tissue specimens (n=93) for immunohistochemistry were purchased as microarray (Outdo Biotech Co., Shanghai, China). All specimens were frozen immediately, stored at liquid nitrogen. The median age of the patients of the 143 ESCC tissues for immunochemistry was 60 years (range, 29–84 years).

### Protein extraction and quantification

Approximately 1×10^7^ cells were grown to 80% confluence and washed six times in 1X phosphate-buffered saline (PBS). Soluble proteins were extracted with lysis (50 mM Tris-HCl pH 7.4, 150 mM NaCl, 1% Triton X-100, 0.1% SDS) with protease inhibitor cocktail (2.5 μM AEBSF, 0.04 μg/ml aprotinin, 0.04 μg/ml leupeptin, 1 mM EDTA ) by super-sonication and followed by centrifugation at 12,000 g for 15 min. Protein concentration was measured by the Bradford method.

### Two dimensional electrophoresis

The soluble proteins from individual tissue specimen and the pooled tissue samples were separated by two dimensional electrophoresis (2-DE). Commercial IPG strips (pH 3–10NL, 18 cm; Amersham Biosciences, Uppsala, Sweden), were rehydrated overnight with 450 μl solution containing 8 M urea, 2% w/v CHAPS, 20 mM DTT, 0.5% v/v IPG buffer, 0.002% bromophenol blue and 1000 μg protein. Electrofocusing was carried out for 60 kVh at 20°C following the manufacturer’s instruction. Prior to the second dimension, the IPG strips were equilibrated for 30 min with 50 mM Tris-HCl pH 8.8, 6 M urea, 30% v/v glycerol, 2% w/v SDS, and a trace of bromophenol blue followed by reduction with 1% of DTT and alkylation with 2.5% of iodoacetamide. The IPG strips were placed into 12% SDS-polyacrylamide gels (26×20 cm) and were further electrophoresed by an EttanII-DE system (Amersham Biosciences) with a programmable power control, 0.5 h at 0.5 W per gel, then at 15 W per gel until the dye front reached the gel bottom. The separated proteins were visualized by Coomassie Brilliant Blue staining.

### Protein identification

Briefly, images of the stained gels were acquired with an Image Scanner (Amersham Biosciences) using transmissive light. The gel images were first analyzed by eyes and subsequently were analyzed by ImageMaster 2D Elite 4.01 (Amersham Biosciences). Protein spots with signals differential intensity reaching 2.0 in 2D gels were excised, then were digested in modified trypsin solution with a final substrate-to-trypsin ratio of 40:1 (W:W) (in 25 mM ammonium bicarbonate). The digested peptides from 2-DE gel spots were analyzed by MALDI-TOF-MS using an Ettan-MALDI-TOF system (Amersham Biosciences). Monoisotopic peptide masses obtained from MALDI-TOF were used to search the NCBInr protein database using Mascot algorithm.

### Immunohistochemistry

For immunohistochemical staining multiple tissue arrays (MTA) of formalin-fixed and ESCC and their matched adjacent normal tissues were incubated with stathmin mAb (3352, Cell Signaling Technology, UK) or control IgG. After washing with 1X PBS, slides were reacted with the biotin-labeled second antibody and then visualized using an ultrasensitive streptavidin-peroxidases system (Maxim Biotech, Fuzhou, China). Semi-quantitative criteria of the stathmin immunoreaction were modified according to previous publications (17,18). Immunostaining was scored as follows: 0, negative; 1, weak; 2, moderate; and 3, strong staining. The percentage of stathmin staining area was graded as 0, no positive staining; 1, <10%; 2, 10–50%; or 3, 50–100%. The staining index was calculated as the multiples of staining intensity and staining area, as described (18,19) The staining index <1 was considered as negative, while 1–4 as weak and >4 as strong.

### Western blot analysis

Western blotting was performed as previously described (20). In briefly, protein extracted from cell lines and tissues specimens were separated using 12% SDS-PAGE, then transferred to polyvinylidene fluoride (PVDF) membranes (Millipore, Bedford, MA). Membranes were blocked by 10% skim milk in 1X PBS. The membranes were incubated with the primary antibodies against stathmin (ab52630, Abcam, UK) or β-actin (Cat. No. A-5316, Sigma, MO) in suitable dilutions. Secondary antibodies were anti-rabbit IgG and anti-mouse IgG, respectively. Signals were detected by chemiluminescence using the ECL kit.

### siRNA transfection

The double-strand small interfering RNAs (siRNAs) were synthesized in duplex and purified forms using Genechem Co. (Shanghai, China). siRNAs targeting stathmin [5′-GAAACGAGAGCACGAGAAAtt-3′ (forward) and 5′-UUUCUCGUGCUCUCGUUUCtt-3′ (reverse)] and non-specific scramble siRNA oligonucletide sequences [5′-UUC UCCGAACGUGUCACGUtt-3′ (forward) and 5′-ACGUGAC ACGUUCGGAGAAtt-3′ (reverse)] were transfected separately into KYSE30 and KYSE410 cells using Lipofectamine 2000 (Invitrogen, CA, USA) according to manufacturer′s protocol. After 72 h, proteins were extracted.

### In vitro wound-healing assay

KYSE30, KYSE410 or EC0156 cells were incubated overnight yielding confluent monolayer for wounding. Wounds were scratched using a pipette tip, photographs were taken immediately (time 0 h) and 24 h after wounding. The distance migrated by the cell monolayer to close the wounded area during this time period was measured. Photos were taken by Leica DMCI microscope (Leica, German) at ×100 magnification.

### Statistical analysis

Data were analyzed by SPSS 16.0 (SPSS Inc., Chicago, IL, USA). χ^2^ test was used to analyze the relationship between stathmin expression with clinicopathologic characteristics. P-values <0.05 were considered as statistically significant.

## Results

### The identification of stathmin in ESCC tissues

**Figure 1:**
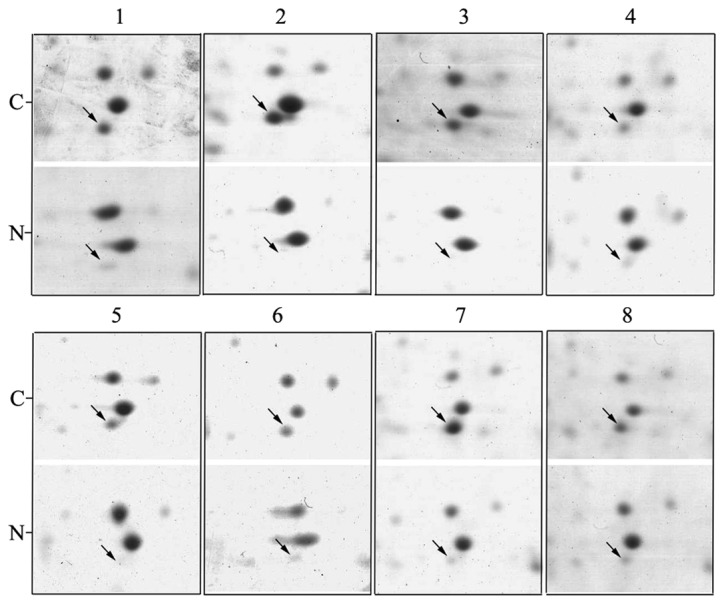
2-DE images of spots identified as stathmin in 8 matched ESCC samples. N, the adjacent normal esophageal tissue; C, ESCC samples. The numbers represent the cases studied.

Firstly, we analyzed differientially expressed proteins using proteomic method between tumor and corresponding normal tissues in ESCC. Results showed stathmin was detected in all 2-DE gels of the 8 pairs of ESCC tissues (Fig. 1), as an obvious differentially expressed spot.

### Stathmin is overexpressed in ESCC tissues

**Figure 2:**
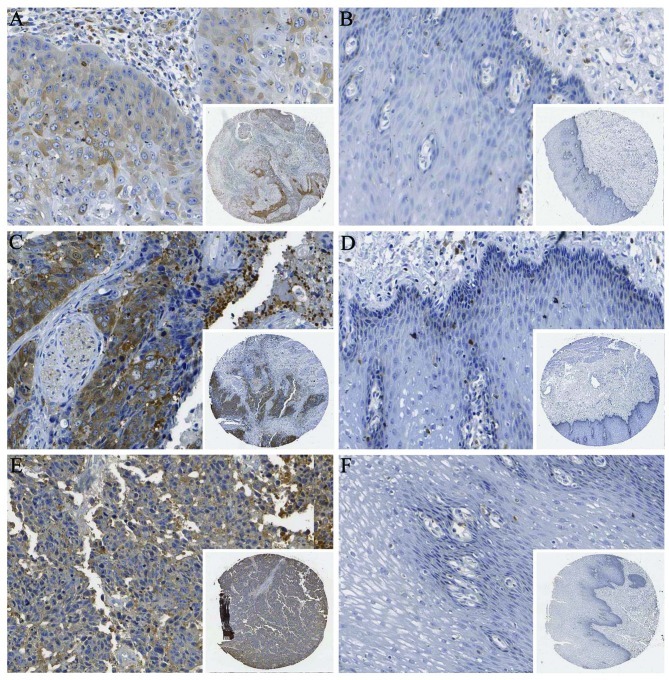
Representative immunohistochemistry staining of stathmin in ESCC specimens. (A, C and E) representative immunohistochemistry staining of cancer (x40), the lower right panels are shown at high magnification (x400), (A) well differentiated, (C) moderately differentiated, (E) poorly differentiated (B, D and F) representative immnohistological staining of normal (x40). The lower right panels are shown high magnification (x400).

Subsequently, we employed immunohistochemistry to analyze the expression of stathmin in 143 ESCC tissue microarray using the anti-stathmin antibody. Strong staining was seen in ESCC tissues, however, the normal tissues were weaker or negatively stained (Fig. 2). The strong staining of stathmin in cancer was 70.63% (101/143), while normal in 27.97% (40/143). In addition, the weak staining of stathmin in cancer was 20.28% (29/143), while normal in 15.38% (22/143). There was a significantly different staining mode between cancer and normal (P<0.05). Statistical analysis showed that overexpression of stathmin was significantly correlated with histological grade (P<0.05) (Table I). However, no correlation was found between stathmin expression and age, gender and lymph node metastasis (P>0.05) (Table I).

### siRNA-mediated reduction in stathmin expression resulted in impaired cell migration

**Figure 3:**
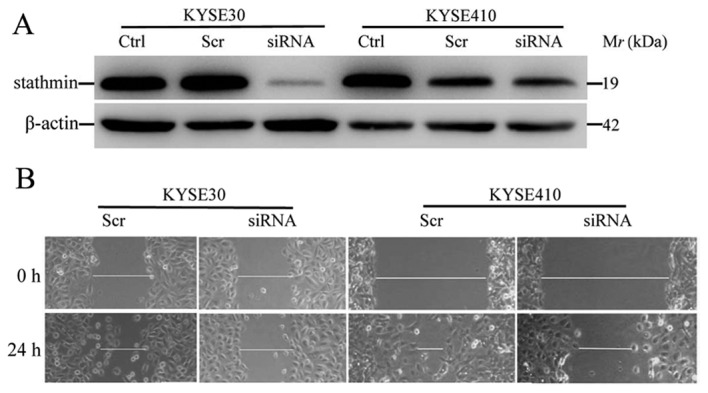
siRNA of stathmin in KYSE30 and KYSE 410 impair cell migration. (A) Analysis the effect of stathmin knockdown in KYSE30 and KYSE 410 using western blot analysis. (B) The wound-recovery result after knockdown in KYSE30 and KYSE410. Microtip scrape of the cultured cells, was then visualized under a microscope at ×100 magnifcation. Ctrl, control; Scr, scramble.

We chose two ESCC cell lines (KYSE30 and KYSE410) as a model. SiRNA was employed to knockdown *stathmin,* and western blotting to detect the effect of siRNA. The results showed that the expression of stathmin was reduced after transfection of siRNA oligonucleotide for 72 h in KYSE30 and KYSE410 (Fig. 3A). Subsequently, wound-healing assay showed that the speed of wound recovery of siRNA *stathmin* was much slower than the scramble in both KYSE30 and KYSE410 (Fig. 3B). The results revealed that cell migration was impaired when deficient of stathmin.

### The phosphorylation of stathmin reduced the motility of EC0156

Considering phosphorylation had been closely associtated with the function of stathmin, we wondered whether stathmin phosphorylation has influence on ESCC cell lines. We selected EC0156 for the next study.

**Figure 4:**
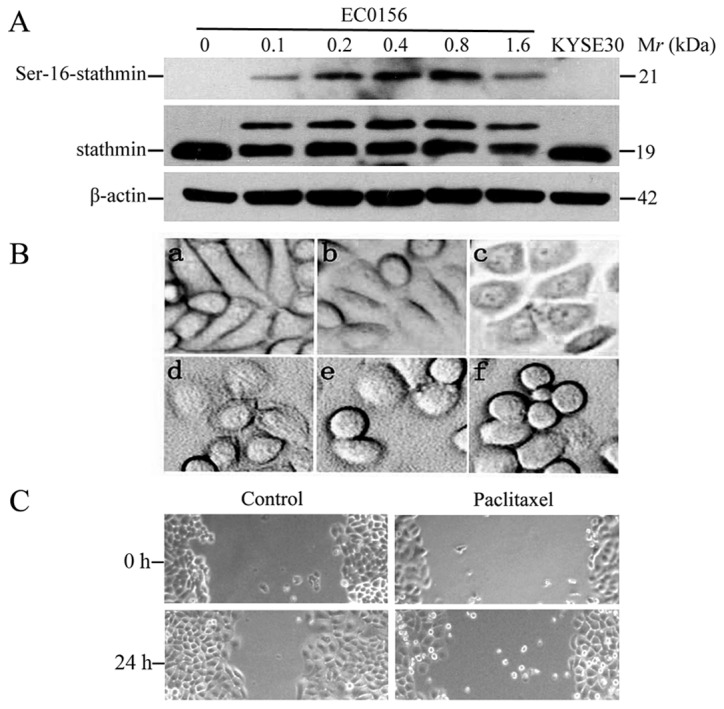
The phosphorylation of stathmin induced by paclitaxel impaired migration in EC0156. (A) Analysis the expression of stathmin in KYSE30 and KYSE 410 using western blot analysis after paclitaxel treatment. The treated paclitaxel dosage in EC0156 was 0.1, 0.2, 0.4, 0.8 and 1.6 μg/ml independently. (B) Cytological morphology variation after paclitaxel treatment. The paclitaxel dosage of treating EC0156 was 0.1, 0.2, 0.4, 0.8 and 1.6 μg/ml, independently. (C) The wound-recovery results after paclitaxel treatment in EC0156. The paclitaxel dosage was 0.8 μg/ml.

EC0156 was treated with paclitaxel, a compound extracted from taxus plants, at gradient dosage from 0 to 1.6 μg/ml. The results revealed that the 19 kDa band of stathmin was decreased after paclitaxel treatment, whereas a new band at about 21 kDa gradiently increased in a dose-dependent manner. We confirmed the new band was phosphorylated stathmin at Ser-16. The tendency increased dramatically between 0.1 and 1.6 μg/ml, reaching a peak at a dosage of 0.8 μg/ml. Conversely, KYSE30 and EC0156 without treatment had no changes (Fig. 4A). Besides, rounded cells were observed in most of the EC0156 through microscopic analysis (Fig. 4B). The number of cells appered slightly reduced. Wound-healing assay showed that the speed of wound recovery of EC0156 cells treated with pacitaxel was much slower than the control, suggesting that cell migration was impaired after stabilized phophorylation of stathmin (Fig. 4C).

## Discussion

Previously, we analyzed differientially expressed proteins using proteomic methods between tumor and nomal tissues in ESCC. We found an obviously overexpressed spot in all tumor 2-DE gels, which was identified as stathmin. Stathmin is ubiquitous, highly conserved 19 kDa cytosolic phosphoprotein that regulates microtubule dynamics (21).

We employed immunohistochemistry to detect the expression of stathmin in ESCC tissue microarray. Here we demonstrated strong expression in 77.14% (108/140) of ESCC tissues. In addition, overexpression of stathmin was significantly correlated with histological grade (P<0.05). However, no correlation was found with age, gender and lymph node metastasis (P>0.05). Wang *et al* revealed that the positive rate of stathmin in 75 ESCC samples was 81.3% and the relative contents of stathmin were significantly correlated with the differentiation degree, lymph node metastasis, invasive depth and TNM stage of ESCC (13). In addition, the basic tendency of stronger stathmin staining in ESCC was consistent with our previous study, which only used 13 ESCC samples (22). So far, many studies have demonstrated stathmin to be overexpressed in many human cancers, including mesothelioma tumor (11), malignant pheochromocytomas (23,24), cervical carcinoma (25), primary nasopharyngeal carcinoma (26), gastric cancer (27), hepatocellular carcinoma (28,29), medulloblastoma (30), endometrial cancer (18) and urothelial carcinoma (31). Above all, overexpression of stathmin was significantly correlated with clinical stage, tumor grade and lymph node metastasis in cancers, including cervical carcinoma, nasopharyngeal carcinoma, gastric cancer, hepatocellular carcinoma and endometrial cancer (18,25–30). Moreover, stathmin may be regarded as a survival prognosis factor in ovarian cancer (32), endometrial cancer (18) and urothelial carcinoma (31). Our previous work, although the ESCC cases were only 13 for immunohistochemistry to analyze the expression of stathmin, we still found the expression of stathmin was overexpressed in ESCC tissue, but there was no correlation with tumor grades (22). In the present study, we expanded the ESCC case numbers for immunohistochemitry. Finally, we found overexpression of stathmin was significantly correlated with histological grade. However, the relationship between stathmin expression and lymph node metastasis did not replicate. There may be two reasons. The limited cases with lymph node metastasis in tissue microarray may lead to information being lost in our study. Another factor worth considered is the tiny difference of the pathologic criteria. In addition, the case number of the present study was much greater than the previous reports in ESCC.

We demonstrated that the speed of wound recovery in stathmin knockdown cells was much slower than the scramble in both KYSE30 and KYSE410 cells, suggesting cell migration was impaired when deficient of stathmin. Similar report exists in gastic cancer, in which cell invasion was significantly reduced by stathmin siRNA in the Matrigel invasion assay (27). Stathmin depletion with siRNA caused significant inhibition of lamellipodia formation, which directed by Pak1-WAVE2-kinesin complex (33). The lamellipodia formation may promote cell migration and invasion. Thus, stathmin depletion might inactivate lamellipodia formation leading to reduction of cell migration.

We demonstrated that the band at 19 kDa of stathmin was decreased when EC0156 was treated by paclitaxel. Meantime, a new band at about 21 kDa appeared in a dose-dependent manner. Subsequently, the new band was confirmed as phosphorylated stathmin at Ser-16. The basic function of stathmin was closely associated with its phosphorylated state. The stathmin-tubulin interaction was dependent on phosphorylation of stathmin (34). Rapidly switching phosphorylation of stathmin regulated microtubule assembly (21). Phosphorylation at Ser-16 and Ser-63 strongly reduced stathmin-tubulin complex formation (35). The effects of stathmin on dynamic instability were strongly attenuated by phosphorylation at Ser-16-and Ser-63 (6). Many protein kinases, including CDC2 (36), MAP (37), Auro B (38) and BGLF4 (39), can phosphorylate stathmin. Stathmin is known to undergo phosphorylation at Ser16 upon cell stimulation, such as paclitaxel at low concentration (40). Paclitaxel is an anticancer drug which interferes with microtubules (41). In the present study, we found paclitaxel may induce stathmin phosphorylation at Ser-16 and influenced the function of stathmin.

Through microscopic analysis, rounded cells were observed in most of EC0156 after the treatment with paclitaxel. The total number EC0156 appeared slightly reduced. Besides, the wound recovery speed of EC0156 treated with paclitaxel was diminished compared to the control, suggesting that cell migration may be impaired after stathmin phophorylation. It is possibly that stabilized phosphorylation of stathmin may disrupt microtubule dynamics and finally impaired the motility of EC0156, which were supported by other reports. Migration can be viewed as a repeated sequences of events that include formation of pseudopodia protrusions, attachment and translocation of the cell body in the direction of the new adhesion sites. The microtubule dynamics instability is important to generate an asymmetrical microtubules array (42). Stronger or long time stabilized phosphorylation of stathmin may impair the microtuble dynamics. Cernuda-Morollon *et al* found that T-cell receptor (TCR)-induced stathmin phosphorylation prevented T cell polarization on ICAM-1 by increasing Rac1 activity and reduced the migration of T cell and changed microtubule dynamics leading to loss of migratory polarity (43). Di Paolo *et al* revealed that phosphorylation at three sites (Ser-16, Ser-25, Ser-63) completely inhibited the tubulin binding capacity of stathmin (35). This phenomenon was also observated in cancer cells. Belletti *et al* revealed that Ser-16 phosphorylation of stathmin enhanced sarcoma cell adhesion and inhibited sarcoma cell motility by mutant methods (44). The present study revealed that paclitaxel may act as a stimulus factor to induce the phosphorylaiton of stathmin leading to impairment of microtubule dynamics. Our observations provide new evidence for understanding the interaction between stathmin phosphorylation and microtubules.

Our observation at cell level was not in accordance with immunohistochemistry analysis. Except for information lost or cancer diversity, different microenvironment between cultured cells and tissues may also contribute. In summary, stathmin was overexpressed in ESCC tissues and overexpression of stathmin was significantly correlated with histological grade. In addition, deficiency or stabilized phosphorylation of stathmin both impaired the migration of ESCC cells. The present study might highlight the potential of stathmin in the therapy and diagnosis of ESCC.

## Figures and Tables

**Table I:** The correlation between stathmin expression and clinicopathological characteristics in ESCC specimens.

		Stathmin			
			
Characteristics	All cases (n) (%)	Negative (%)	Weak (%)	Strong (%)	P-value
Age					>0.05
≥60	80 (55.94)	7 (8.75)	14 (17.50)	59 (73.75)	
<60	63 (44.06)	6 (9.52)	15 (23.81)	42 (66.67)	
Gender					>0.05
Male	108 (75.52)	11 (10.19)	24 (22.22)	73 (67.59)	
Female	35 (24.48)	2 (5.71)	5 (14.29)	28 (80.00)	
Tumor grade					0.019
Well differentiated	36 (25.17)	8 (22.22)	8 (22.22)	20 (55.56)	
Moderately differentiated	80 (55.94)	5 (6.25)	16 (20.00)	59 (73.75)	
Poor differentiated	27 (18.89)	0 (0.00)	5 (18.52)	22 (81.48)	
Lymph node metastasis^a^					>0.05
Present	38 (40.43)	3 (7.90)	3 (7.90)	32 (84.20)	
Not present	56 (59.57)	2 (3.57)	7 (12.50)	47 (83.93)	

a Only 94 cases have lymph node information.
